# Leptin mutation and mycobacterial infection lead non-synergistically to a similar metabolic syndrome

**DOI:** 10.1007/s11306-022-01921-8

**Published:** 2022-08-07

**Authors:** Yi Ding, Mariëlle C. Haks, Susan J. F. van den Eeden, Tom H. M. Ottenhoff, Amy C. Harms, Thomas Hankemeier, Muhamed N. H. Eeza, Jörg Matysik, A. Alia, Herman P. Spaink

**Affiliations:** 1 Institute of Biology, Leiden University, Sylviusweg 72, 2333 BE Leiden, The Netherlands; 2 Department of Infectious Diseases, Leiden University Medical Center, Leiden, The Netherlands; 3 Leiden Academic Centre for Drug Research, Leiden University, Leiden, The Netherlands; 4 Institute of Analytical Chemistry, University of Leipzig, Leipzig, Germany; 5 Institute for Medical Physics and Biophysics, University of Leipzig, Leipzig, Germany; 6 Leiden Institute of Chemistry, Leiden University, Leiden, The Netherlands

**Keywords:** Metabolomics, Transcriptomics, Tuberculosis, Leptin mutant zebrafish, *Ob/ob* mice, Non-synergy

## Abstract

**Introduction:**

The leptin signaling pathway plays an important role as a key regulator of glucose homeostasis, metabolism control and systemic inflammatory responses. However, the metabolic effects of leptin on infectious diseases, for example tuberculosis (TB), are still little known.

**Objectives:**

In this study, we aim to investigate the role of leptin on metabolism in the absence and presence of mycobacterial infection in zebrafish larvae and mice.

**Methods:**

Metabolites in entire zebrafish larvae and the blood of mice were studied using high-resolution magic-angle-spinning nuclear magnetic resonance (HR-MAS NMR) spectroscopy and mass spectrometry, respectively. For transcriptome studies of zebrafish larvae, deep RNA sequencing was used.

**Results:**

The results show that leptin mutation leads to a similar metabolic syndrome as caused by mycobacterial infection in the two species, characterized by the decrease of 11 amine metabolites. In both species, this metabolic syndrome was not aggravated further when the leptin mutant was infected by mycobacteria. Therefore, we conclude that leptin and mycobacterial infection are both impacting metabolism non-synergistically. In addition, we studied the transcriptomes of *lepb*^*ibl54*^ mutant zebrafish larvae and wild type (WT) siblings after mycobacterial infection. These studies showed that mycobacteria induced a very distinct transcriptome signature in the *lepb*^*ibl54*^ mutant zebrafish compared to WT sibling control larvae. Furthermore, *lepb*^*ibl55*^* Tg* (*pck1:luc1*) zebrafish line was constructed and confirmed this difference in transcriptional responses.

**Conclusions:**

Leptin mutation and TB lead non-synergistically to a similar metabolic syndrome. Moreover, different transcriptomic responses in the *lepb*^*ibl54*^  mutant and TB can lead to the similar metabolic end states.

**Supplementary Information:**

The online version contains supplementary material available at 10.1007/s11306-022-01921-8.

## Introduction

Tuberculosis (TB) is an infectious disease which caused around 10 million cases and 1.2 million deaths reported to the World Health Organization (WHO, 2020). Approximately one quarter of the world’s population is latently infected with *Mycobacterium tuberculosis* (*Mtb*), the causative agent of TB (WHO, 2020). In around 5–10% of these cases, latent infection progresses to active TB (Kiazyk & Ball, 2017). TB is often associated with severe wasting syndrome accompanied by loss of whole body mass and increased risk of death (Paton & Ng, 2006). The metabolic abnormalities underlying the wasting syndrome in TB have been studied in humans and TB animal models (Ding et al., 2020; Weiner et al., 2018). TB causes metabolic reprogramming characterized by decrease of many metabolites in the blood of patients from Africa (Weiner et al., 2018), China (Deng et al., 2021), Indonesia (Vrieling et al., 2019) and the Netherlands (Ding et al., 2020). The metabolic responses towards mycobacterial infection in the blood of mice were highly similar to that in TB patients (Ding et al., 2020). We have shown that even in entire zebrafish larvae, infection with *Mycobacterium marinum* (*M. marinum*), a natural fish pathogen and a close relative of *Mtb*, leads to a very similar metabolic syndrome as observed in mice and patients after infection with *Mtb* (Ding et al., 2020). Ten metabolites were identified as common biomarkers of mycobacterial infection in TB patients, mice and zebrafish larvae (Ding et al., 2020).

One of the risk factors for the development of TB is type 2 diabetes mellitus (T2DM; Dooley & Chaisson, 2009). T2DM patients are three times more likely to develop progressive TB infection than normoglycemic people (Restrepo Blanca & Schlossberg, 2016). TB accompanied by T2DM leads to higher *Mtb* bacillary loads in sputum compared with TB patients without T2DM (Andrade et al., 2014). This might be due to the defects in the immune responsiveness in diabetic patients (Ronacher et al., 2015). Alternatively, the changes in system metabolism associated with T2DM could lead to a higher risk of TB (Salgado-Bustamante et al., 2018). Interestingly, both TB and T2DM can lead to a similar metabolic syndrome that is accompanied by muscle wasting (Salgado-Bustamante et al., 2018; Vrieling et al., 2018). Mouse and zebrafish mutants in leptin signaling genes are used to study metabolic alterations associated with T2DM (Giesbertz et al., 2015; He et al., 2021; Michel et al., 2016; Tups et al., 2017). These studies have shown that leptin, in addition to its function in controlling food intake, plays an evolutionary conserved role in regulating glucose homeostasis (Michel et al., 2016; Tups et al., 2017). Leptin has also been shown to have a function in mediating a glucose-fatty acid cycle to maintain glucose homeostasis under starvation condition in rats (Perry et al., 2018). We previously found that leptin deficiency causes similar metabolite alterations in both mice and zebrafish larvae (Ding et al., 2021). These metabolic changes show similar features as observed during progression of TB in human patients, mice and zebrafish larvae (Ding et al., 2021). Studies in a mouse leptin mutant have provided evidence that leptin plays a role in the early immune response to *Mtb* infection (Wieland et al., 2005). Several studies have shown a correlation between the serum level of leptin and the risk of acquiring active TB (Mansour et al., 2019; Soh et al., 2021; van Crevel et al., 2002; Ye & Bian, 2018). The function of leptin in the susceptibility of TB and T2DM is linked to the important role in leptin as a major player in inflammatory processes and to its function as a regulator of system metabolism (Pérez-Pérez et al., 2020; Vrieling et al., 2019). However, the connections between the mechanisms underlying the role of leptin in TB and T2DM are still unknown.

In this study, we investigated the metabolic response in a leptin mutant in the absence and presence of mycobacterial infection in mice and zebrafish larvae. We compared the effect of mycobacterial infection in the leptin mutant zebrafish larvae and mice using metabolomics. Our results show that leptin mutations and mycobacterial infection lead to a similar metabolic syndrome. This metabolic syndrome, however, did not increase further in severity after mycobacterial infection in the leptin mutant. Subsequent transcriptome studies in zebrafish larvae showed that mycobacteria induced a very distinct transcriptome signature in the leptin mutant compared to the wild type (WT) sibling control. Apparently, different transcriptomic responses can lead to the same metabolic end states. Therefore, we conclude that leptin and mycobacterial infection control metabolism in different ways despite shared metabolic features.

## Materials and methods

### Mice

Male *ob/ob* mice and lean C57BL/6 WT mice were obtained from Charles River Laboratories. Eight mice per group were nasally infected with 10^5^ CFU of *Mtb* strain H37Rv and another eight mice per group were mock infected at 6-week of age. The mice were kept under standard BSLIII conditions for 8 weeks in the animal facility of the Leiden University Medical Center (LUMC). Male mice were chosen because metabolic variation due to the hormonal cycle is limited. The mice were kept on a standard-chow diet with ad libitum access to food and water. One *ob/ob* mouse and one WT infected mouse had to be sacrificed at an early stage due to malocclusion. The mice were sacrificed at week 14 and blood, lung and spleen were collected. Mouse serum samples were collected from clotted blood tubes and mixed with pre-heated 80% ethanol at a 1:3 ratio (end concentration: 60% ethanol) in polypropylene screwcap tubes. Samples were heated for 10 min at 90 °C and subsequently chilled on ice for 10 min before centrifugation at 13,000 rpm for 10 min at 4 °C. Supernatants were harvested and stored at − 80 °C for LC–MS analysis. Handling of mice was conducted in compliance with European Community Directive 86/609 for the Care and Use of Laboratory Animals and in accordance with the regulations set forward by the LUMC Animal Care Committee.

### Zebrafish larvae

Zebrafish were handled in compliance with the local animal welfare regulations and maintained according to standard protocols (http://zfin.org). Zebrafish breeding and embryo collection were performed as described previously (Avdesh et al., 2012). Two mutant *lepb*^*ibl54*^ and *lepb*^*ibl55*^ and WT sibling *lepb*^+^ zebrafish lines were generated, screened and raised as described previously (He et al., 2021). The *lepb*^*ibl54*^ mutant line with a 7 base pair deletion encompassing TAGAGGG in exon 2 was used for high-resolution magic-angle-spinning nuclear magnetic resonance (HR-MAS NMR) measurement and deep sequencing studies. The other *lepb*^*ibl55*^ mutant with a 8 base pair deletion encompassing TAGAGGGC in exon 2 was used for the *pck1* luciferase reporter assay and is considered equivalent as there are no observable differences in the phenotypes such as differences in the blood glucose level between two *lepb* adult mutants (He et al., 2021). Zebrafish *lepb*^+^ and *lepb*^*ibl54*^ embryos were collected from 6-month-old WT and *lepb*^*ibl54*^ mutant parents, respectively. The embryos were injected into yolk with *M. marinum* strain M labelled with mWasabi plasmid pTEC15 vector22 or mock injected with 2% polyvinylpyrrolidone 40 (pvp) at 4 to 6 h post fertilization (hpf). *M. marinum* preparation were followed by the protocol of a previous study (Benard et al., 2012). Microinjection of zebrafish embryos was performed using an automatic microinjection system (Life Science Methods, The Netherlands) described previously (Spaink et al., 2013). Zebrafish larvae at 5 days post fertilization (dpf) were collected and stored at −80℃ until further analysis. For HR-MAS NMR measurement, 3 replicates of 120 pooled larvae were used and each sample was measured three times to avoid technical issues.

### LC–MS/MS

Metabolite levels in mice serum were measured in individual replicates using a targeted LC–MS/MS platform as described before (Ding et al., 2020). Samples were randomized and run in one batch which included a calibration line, QC samples and blanks. QC samples were analyzed every 10 samples. They were used to assess data quality and to correct for instrument responses.

The amine platform covers amino acids and biogenic amines employing an Accq-Tag derivatization strategy adapted from a previously published protocol (Noga et al., 2012). Briefly, 5.0 μL of each sample was spiked with an internal standard solution. Then proteins were precipitated by the addition of MeOH. The supernatant was dried in a speedvac. The residue was reconstituted in borate buffer (pH 8.5) with AQC reagent. 1.0 μL of the reaction mixture was injected into the UPLC–MS/MS system. Chromatographic separation was achieved by an Agilent 1290 Infinity II LC System on an Accq-Tag Ultra column. The UPLC was coupled to electrospray ionization on a triple quadrupole mass spectrometer (AB SCIEX Qtrap 6500). Analytes were detected in the positive ion mode and monitored in Multiple Reaction Monitoring (MRM) using nominal mass resolution. Acquired data were evaluated using MultiQuant Software for Quantitative Analysis (AB SCIEX, Version 3.0.2). The data are expressed as relative response ratios (target area/ISTD area; unit free) using proper internal standards. For analysis of amino acids, their ^13^C^15^N-labeled analogs were used. For other metabolites, the closest-eluting internal standard was employed. After quality control correction, metabolite targets complied with the acceptance criteria of RSDqc < 15%. Using this platform, we were able to identify 41 metabolites in blood samples from mice.

### MS data analysis

Data was analyzed using the software package MetaboAnalyst 5.0 (Pang et al., 2021). MetaboAnalyst offers the possibility to provide automated data reports which we used for archiving data sets. Default settings were used with log transformation and auto scaling of the data for normalization. Naming of the metabolites is based on reference compounds using standard nomenclature of the human metabolome database (https://hmdb.ca/).

### ^1^H HR-MAS NMR measurement of intact zebrafish larvae

Metabolic profiling by ^1^H HR-MAS NMR spectroscopy was performed as described in a previous study (Ding et al., 2021). Zebrafish larvae were carefully transferred to a 4-mm zirconium oxide MAS NMR rotor (Bruker BioSpin GmbH, Germany). As a reference (^1^H chemical shift at 0 ppm), 10 µL of 100 mM deuterated phosphate buffer (KD_2_PO4, pH 7.0) containing 0.1% (w/v) trimethyl-silylpropanoic acid (TSP) was added to each sample. The rotor was then placed immediately inside the NMR spectrometer.

All HR-MAS NMR experiments were performed on a Bruker DMX 600-MHz NMR spectrometer, which was equipped with a 4-mm HR-MAS dual inverse ^1^H/^13^C probe with a magic angle gradient and spinning rate of 6 kHz with a proton resonance frequency of 600 MHz. Measurements were carried out at a temperature of 277 K using a Bruker BVT3000 control unit. Acquisition and processing of data were done with Bruker TOPSPIN software 2.1 (Bruker BioSpin GmbH, Germany).

A standard pulse sequence “ZGPR” (from Bruker's pulse program library) with water pre-saturation was used for measuring one-dimensional ^1^H HR-MAS NMR spectra. Each one-dimensional spectrum was acquired applying a spectral width of 12 kHz, time domain data points of 8k, number of averages of 128, an acquisition time of 170 ms and a relaxation delay of 2 s. All spectra were processed by an exponential window function corresponding to a line broadening of 1 Hz and zero-filled before Fourier transformation. NMR spectra were phased manually and automatically baseline corrected using TOPSPIN 2.1 (Bruker BioSpin GmbH, Germany). The total analysis time (including sample preparation, optimization of NMR parameters, and data acquisition) of ^1^H HR-MAS NMR spectroscopy for each sample was approximately 20 min.

### NMR analysis

The one-dimensional ^1^H HR-MAS NMR spectra were corrected for baseline, phase shifts and reference using TOPSPIN 2.1 (Bruker BioSpin GmbH, Germany). Subsequently, the spectra were subdivided in the range between 0 and 10 ppm into buckets of 0.04 ppm using MestReNova software version 11.0 (Mestrelab Research S.L., Santiago de Compostela, Spain). The resulting data matrix was saved as the format of script: NMR CSV matrix (transposed) (*.CSV, *.txt). This was then imported into MetaboAnalyst 5.0 for multivariate analysis using partial least squares discriminant analysis (PLS-DA). Correlation coefficients with *p* < 0.05 were considered statistically significant. Quantification of metabolites was performed using Chenomx NMR Suite 8.6 (Edmonton, Alberta, Canada), which allowed for qualitative and quantitative analysis of an NMR spectrum by fitting spectral signatures from HMDB database to the respective spectrum. Assignment of peaks was based on the chemical shifts of compounds of interest in Chenomx software. Statistical analysis (t-tests) of the NMR quantification results was performed with GraphPad Prism 8.0.1 (San Diego, CA, USA) and *p* < 0.05 were considered significant.

### RNA isolation

Zebrafish larvae from *lepb*^+^ and *lepb*^*ibl54*^ infected and control groups (15 pooled larvae/replicate, n = 3) were resuspended and crushed in 0.5 mL of TRIzol Reagent. Subsequently, total RNA was extracted in accordance with the manufacturer’s instructions. Contaminating genomic DNA was removed using DNase I digestion for 15 min at 37 °C. RNA concentration was determined by NanoDrop 2000 (Thermo Scientific, The Netherlands). RNA integrity (RIN) was assessed by bioanalyzer (Agilent) and samples with RIN values > 6 were used for further library construction and sequencing.

### Deep sequencing of zebrafish larvae

Deep sequencing of the zebrafish larvae was performed by GenomeScan B.V. (Leiden, the Netherlands). The NEBNext Ultra II Directional RNA Library Prep Kit for Illumina (NEB #E7760S/L) was used to process the samples. Briefly, mRNA was isolated from total RNA using oligo-dT magnetic beads. After fragmentation of the mRNA, a cDNA synthesis was performed. This was used for ligation of the sequencing adapters and PCR amplification of the resulting product. The quality and yield after sample preparation was measured with Fragment Analyzer. The size of the resulting products was consistent with the expected size distribution (a broad peak between 300 and 500 bp). Clustering and DNA sequencing using the NovaSeq6000 was performed according to manufacturer's protocols. A concentration of 1.1 nM of DNA was used. For the zebrafish larval samples, data sets of paired end reads of 150 nucleotides were obtained with at least 20 million reads of reads that could be mapped to the zebrafish genome version GRCz11.

### Deep sequencing data mapping and analysis

Sequencing data of zebrafish larvae were aligned and mapped to the zebrafish genome GRCz11 using CLC Genomics, and differential gene expression was analyzed using DESeq2 v1.21.1. Gene ontology (GO) term enrichment and KEGG pathway analysis were performed in DAVID Bioinformatics Resources 6.8 (https://david.ncifcrf.gov/).

### *Pck1* luciferase reporter assay

The *Tg* (*pck1:luc1*) zebrafish line obtained from the laboratory of Dr. Stainier (Gut et al., 2013) was out-crossed with the *lepb*^*ibl55*^ mutant (8 bp deletion) to obtain a heterozygous *lepb*^*ibl55/*+^ *Tg* (*pck1*:luc1) line. Subsequently, the heterozygous adult zebrafish were in-crossed, raised and genotyped for *lepb*^+^*Tg* (*pck1:luc1*) and *lepb*^*ibl55*^ *Tg* (*pck1:luc1*) zebrafish lines. The embryos of the two lines were injected into the yolk with 300 colony-forming unit (CFU) *M. marinum* strain M labelled with mWasabi plasmid pTEC15 vector22 or mock injected with 2% pvp40 at 4 to 6 hpf. Zebrafish larvae at 5 dpf were washed and transferred (one larva/well) into a 96-well flat clear bottom white polystyrene TC-treated microplate (Corning Incorporated, Costar 3610). 100 μL egg water and 100 μL steady-glo (Promega) were added to the wells followed by incubation of 30 min at room temperature to generate a bioluminescence signal. The luminescence signal was then detected by a microplate reader (TECAN, Infinite M1000).

## Results

### Measurement of bacterial burden and metabolic profiles of *lepb* mutant and control zebrafish larvae in the absence and presence of *M. marinum* infection

**Fig. 1:**
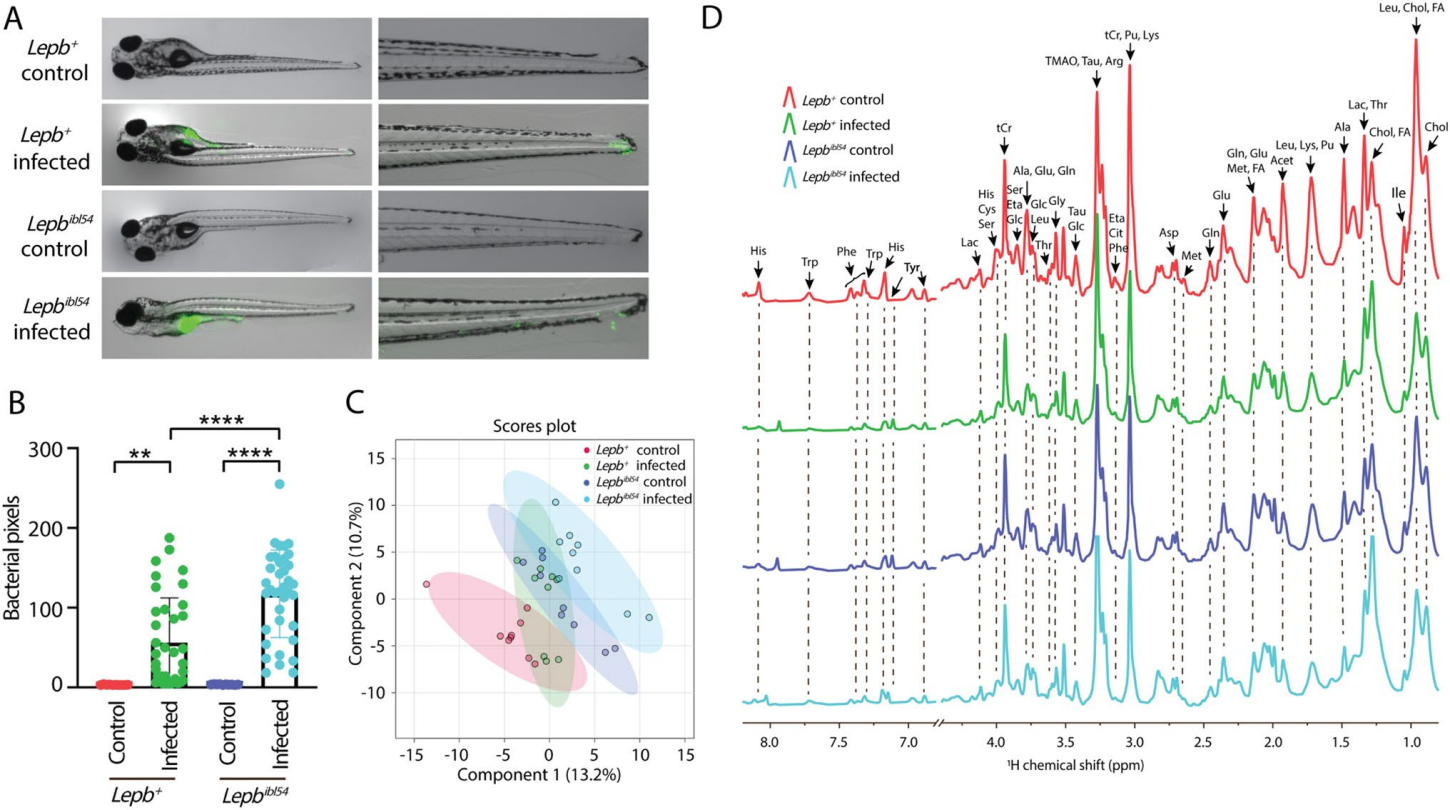
Bacterial loads and metabolic profiles of *lepb*^+^ and *lepb*^*ibl54*^ zebrafish larvae in the absence and presence of *M. marinum* infection. **A** Representative overlap images of bright field and fluorescent images of 5 dpf entire zebrafish larvae (left panel) and the tail part of the larvae (right panel) from the *lepb*^+^ and *lepb*^*ibl54*^ group in the absence and presence of infection. **B** Quantifications of bacterial pixels of the entire larvae in four groups. **p < 0.01, ****p < 0.0001. **C** PLS-DA analysis of metabolic profiles of pooled zebrafish larvae measured by HR-MAS NMR spectroscopy from the four groups. *PLS-DA* partial least square discriminant analysis. **D** The representative HR-MAS NMR spectra of pooled zebrafish larvae from the fours groups. *Acet* acetate, *Ala* alanine, *Arg* arginine, *Asp* aspartate, *Chol* cholesterol, *Cit* citrulline, *Cys* cysteine, *Eta* ethanolamine, *FA* fatty acid, *Glc* glucose, *Gln* glutamine, *Glu* glutamate, *Gly* glycine, *His* histidine, *Ile* isoleucine, *Lac* lactate, *Leu* leucine, *Lys* lysine, *Met* methionine, *Phe* phenylalanine, *Pu* putrescine, *Ser* serine, *Tau* taurine, *Thr* threonine, *tCr* total creatine (creatine + phosphocreatine), *Trp* tryptophan, *Tyr* tyrosine, *NMR* nuclear magnetic resonance

*Lepb* mutant (*lepb*^*ibl54*^) zebrafish larvae and their WT siblings (*lepb*^+^) were injected in the yolk with mWasabi-labeled *M. marinum* strain M or mock-injected with 2% polyvinylpyrrolidone 40 (pvp) at 4 to 6 hpf. Images of the four groups of zebrafish larvae were acquired at 5 days post infection (dpi) and the representative images are shown in Fig. 1A. The images showed that most bacteria after *M. marinum* infection in both *lepb*^+^ and *lepb*^*ibl54*^ larvae were present in yolk but were also detectable in the tail (Fig. 1A). Pixel count quantification of bacterial burden in entire larvae was significantly higher in the *lepb*^*ibl54*^ infected zebrafish larvae than in the *lepb*^+^ siblings (Fig. 1B). However, there is no significant difference of bacterial burden in the tail parts of the two groups. Metabolic profiles of the four groups of pooled zebrafish larvae were measured by HR-MAS NMR spectrometry. A PLS-DA scores plot on the metabolic profiles of zebrafish larvae showed three separate clusters (Fig. 1C). The *lepb*^+^ infected cluster overlapped with the other three clusters (Fig. 1C). The HR-MAS NMR spectra were divided into two major regions: 0.8–4.4 ppm and 6.7–8.2 ppm (Fig. 1D). Peak assignment was performed according to earlier literature (Ding et al., 2021) and the chemical shift of the metabolites in Chenomx NMR Suite 8.6 software which contain information from library of compounds spectra including from the HMDB database (Fig. 1D). The identity of metabolites was additionally confirmed with measurement of two-dimensional homonuclear correlation spectroscopy (^1^H–^1^H COSY) (Supplementary Fig. 1) as described previously (Roy et al., 2017). There were 27 metabolites assigned, including alanine, lysine, lactate and phenylalanine (Fig. 1D). The fold change (FC) and *p* value of all the measured metabolites from zebrafish larvae in different comparisons are shown in Supplementary Table 1.

### Mutation of the *lepb* gene and *M. marinum* infection lead non-synergistically to a similar metabolic syndrome in zebrafish larvae

**Fig. 2:**
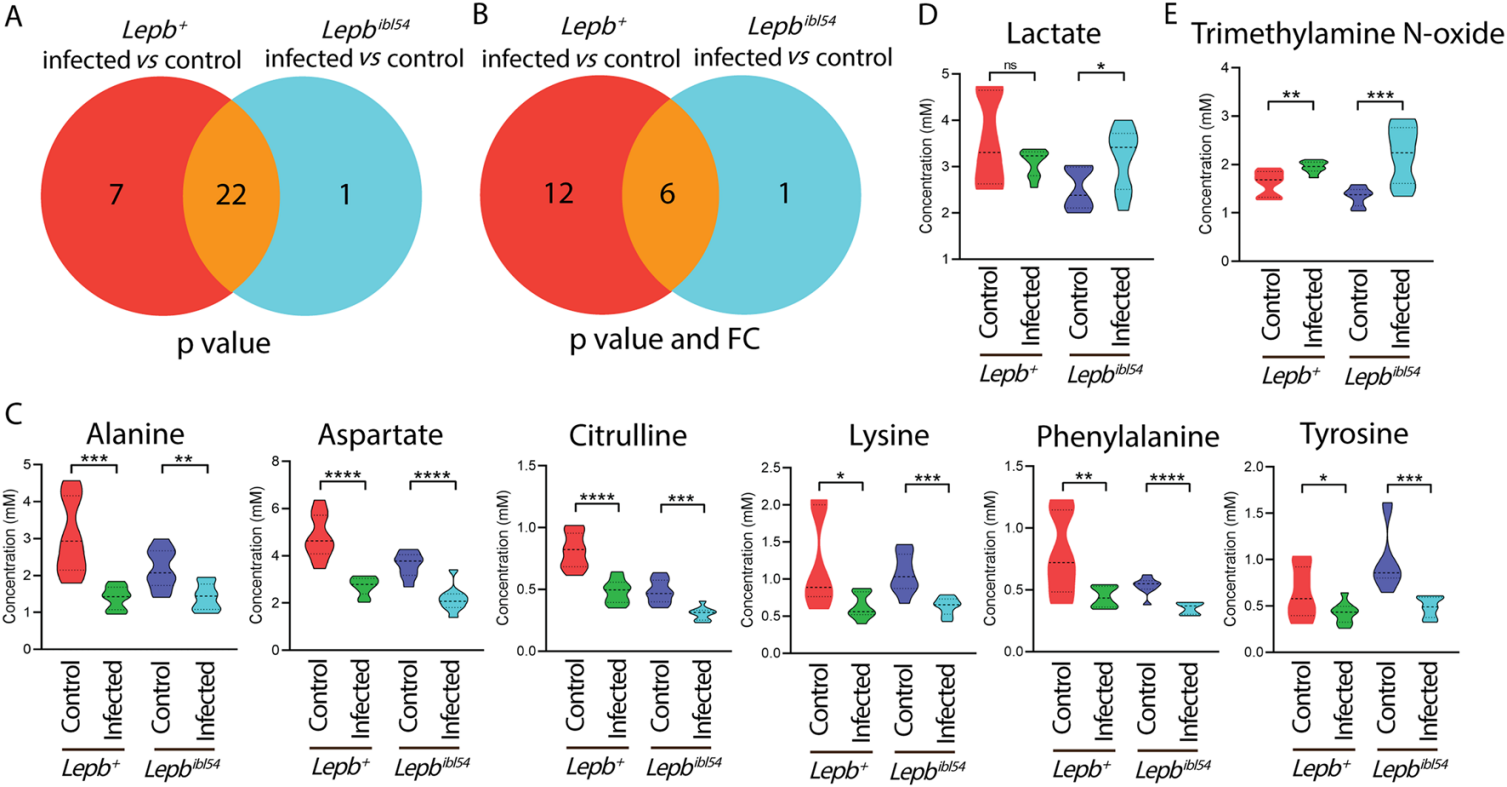
Venn diagrams show the number of metabolites measured by HR-MAS NMR spectroscopy in response to infection in the *lepb*^+^ and *lepb*^*ibl54*^ zebrafish larvae. **A** A Venn diagram shows the number of metabolites in response to *M. marinum* infection in the *lepb*^+^ and *lepb*^*ibl54*^ larvae with *p* < 0.05. **B** A Venn diagram shows the number of metabolites in response to *M. marinum* infection in the *lepb*^+^ and *lepb*^*ibl54*^ larvae with *p* < 0.05 and FC > 1.5 or FC <  − 1.5. *FC* fold change. **C** Quantification of the common six metabolites in **B**. ****p < 0.0001. **D** Quantification of the one metabolite lactate in **A**. *p < 0.05. *ns* non-significant. **E** Quantification of the one metabolite trimethylamine N-oxide in **B**. **p < 0.01, ***p < 0.001

**Fig. 3:**
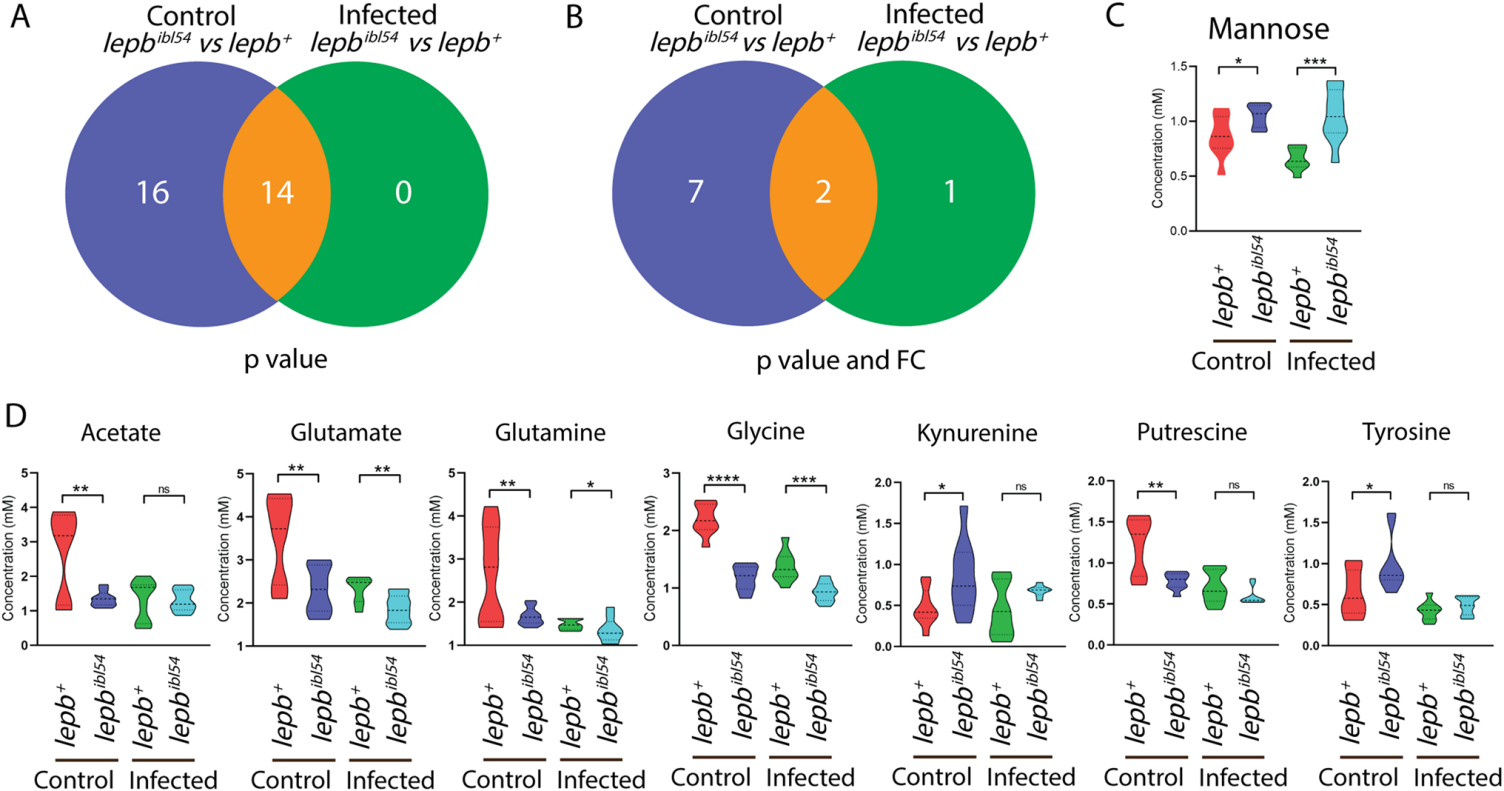
Venn diagrams show the number of metabolites from pooled zebrafish larvae measured by HR-MAS NMR spectroscopy between the *lepb*^*ibl54*^ and *lepb*^+^ in the uninfected control and infected conditions. **A** A Venn diagram shows the number of metabolites between the *lepb*^*ibl54*^ and *lepb*^+^ zebrafish larvae in the uninfected control and infected conditions with *p* < 0.05. **B** A Venn diagram shows the number of metabolites between the *lepb*^*ibl54*^ and *lepb*^+^ zebrafish larvae in the uninfected control and infected conditions with *p* < 0.05 and FC > 1.5 or FC <  − 1.5. *FC* fold change. **C** Quantification of the one metabolite mannose in **B**. *p < 0.05, ***p < 0.001. **D** Quantification of the seven metabolites in **B**. **p < 0.01, ****p < 0.0001. *ns* non-significant

Firstly, we compared the result of the WT *M. marinum* infection in our current HR-MAS NMR study with previously published infection in zebrafish larvae using solution NMR (Ding et al., 2020) (Supplementary Fig. 2). The result showed 20 common metabolites in the two data sets, confirming most of the previously reported biomarkers for infection in zebrafish larvae (Supplementary Fig. 2). Secondly, we generated Venn diagrams to compare the metabolic effect of infection in the *lepb*^*ibl54*^ mutant compared to the WT sibling control at a *p* value < 0.05 with and without applying a 1.5-FC filter. The Venn diagrams of Fig. 2A and 2B showed that the number of metabolites of which the levels were changed after infection in the *lepb*^*ibl54*^ mutant was lower than in the *lepb*^+^ sibling control (Fig. 2C). Only the levels of two metabolites, lactate and trimethylamine N-oxide, were specifically higher in the mutant group after infection while it was lower in the WT after infection (Fig. 2D, E). The Venn diagrams of Fig. 3A, B showed that the number of metabolites of which the levels were different between the *lepb*^*ibl54*^ mutant and WT in the absence of infection was much higher than in the presence of infection. Only mannose had a higher level in the *lepb*^*ibl54*^ than *lepb*^+^ zebrafish larvae after infection (Fig. 3C). As can be seen in Figs. 2C and 3D, this is the consequence of many of the metabolite levels decreasing in response to infection in the WT were already decreased in the absence of infection in the mutant compared to the WT. In conclusion, infection in the *lepb*^*ibl54*^ mutant does not lead to synergistic lowering of the levels of the infection biomarker metabolites. Therefore, we can conclude there is no clear synergy of the effects of *lepb* mutation and *M. marinum* infection on metabolism.

### Metabolic profiles of the blood of leptin mutant *ob/ob* and wild type mice in the absence and presence of *Mtb* infection

**Fig. 4:**
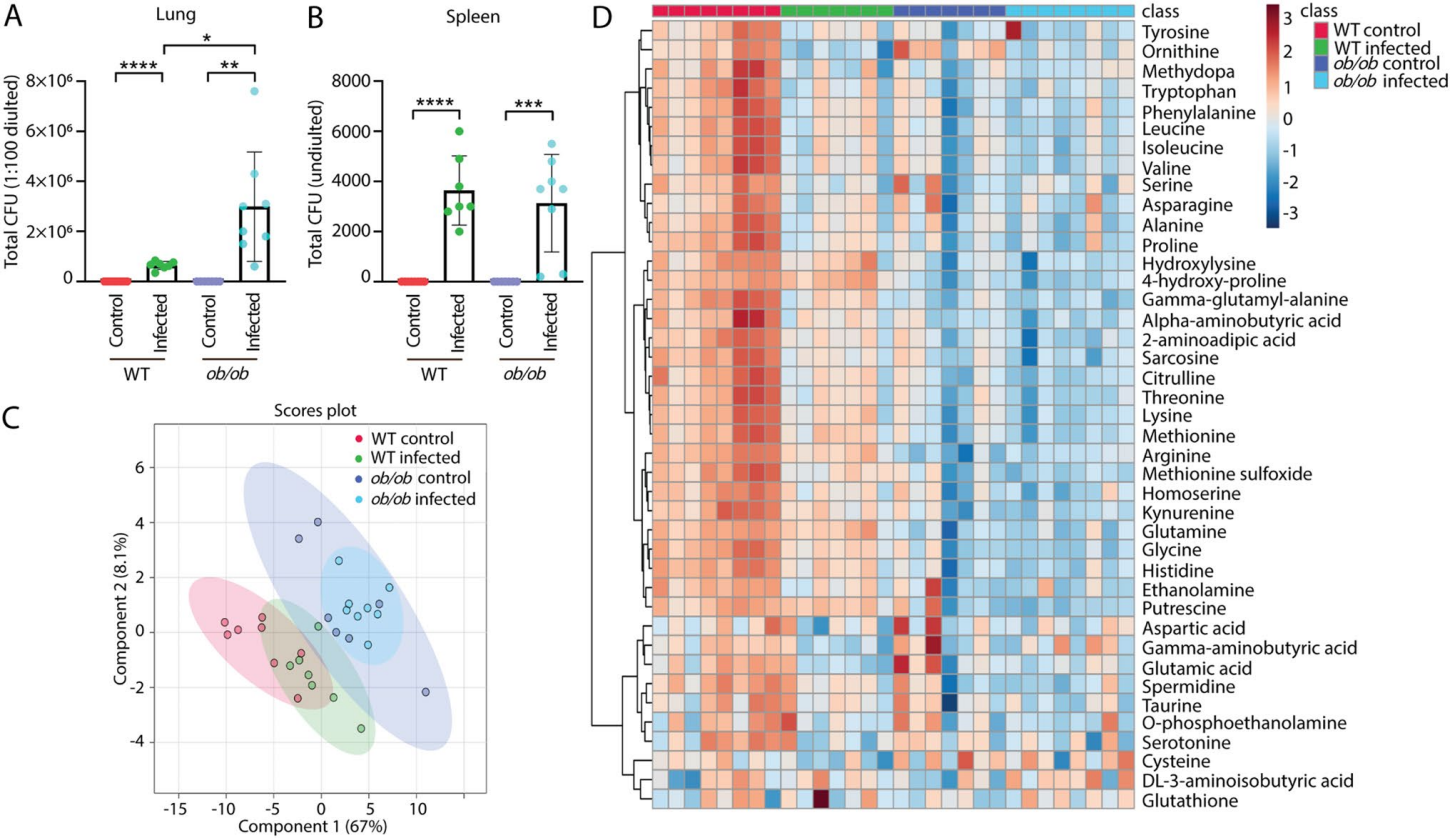
Bacterial loads and metabolic profiles of the blood of wild type and *ob/ob* mice with and without *Mtb* infection. **A** Total CFU (1:100 diluted) of the lungs from the WT and *ob/ob* mice in the absence and presence of infection. *CFU* colony forming unit, *WT* wild type. *p < 0.05, **p < 0.01, ****p < 0.0001. **B** Total CFU (undiluted) of the spleen from the four groups. ***p < 0.001. **C** PLS-DA analysis of the blood metabolic profiles measured by mass spectrometry from the four mouse groups. *PLS-DA* partial least square discriminant analysis. **D** Heatmap analysis of the blood metabolic profiles measured by mass spectrometry from the four mouse groups

Leptin deficient *ob/ob* mice and lean C57BL/6 mice, as a WT control, were intranasally infected with *Mtb*. After 8 weeks, the lungs and spleens were collected and analyzed for bacterial CFU. Plating of bacteria from the isolated lung and spleen materials showed that the mice were systemically infected by *Mtb* in both *ob/ob* and WT mice (Fig. 4A, B). There was more infection in the lungs of *ob/ob* mice than those of WT mice, but not in the spleen (Fig. 4A, B). The metabolic profiles of the blood of these mice were measured by mass spectrometry (MS). A PLS-DA scores plot of the blood metabolic profiles showed that the data sets of the *ob/ob* and WT mice could be separated based on two principal components (Fig. 4C). However, the control and infected data sets were not completely separated in the *ob/ob* and WT mice (Fig. 4C). A heatmap analysis of the blood metabolic profiles showed the abundances of 41 metabolites which were significantly changed in the comparison of infected versus uninfected in the two groups of mice (Fig. 4D). It reveals that the levels of a majority of those metabolites were reduced in the WT mice after *Mtb* infection (Fig. 4D). The levels of the metabolites in *ob/ob* mice were not obviously altered due to infection (Fig. 4D), consistent with the above observations in the zebrafish larvae model. The FC and *p* value of all the measured metabolites from blood of mice in different comparisons are shown in Supplementary Table 2.

### Mutation of the leptin gene and *Mtb* infection lead non-synergistically to a similar metabolic syndrome in mice

**Fig. 5:**
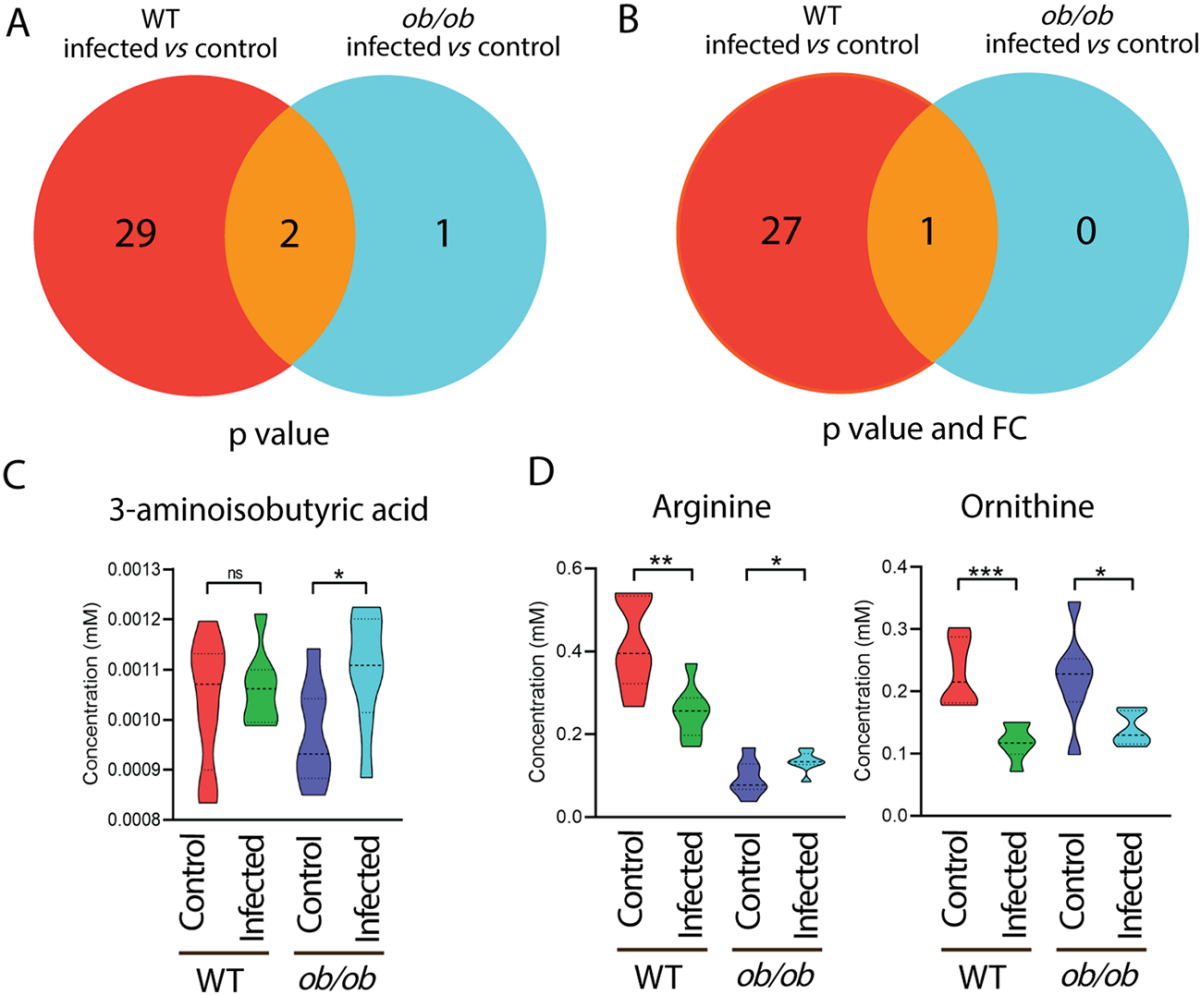
Venn diagrams show the number of metabolites measured by mass spectrometry in response to infection in the blood of wild type and *ob/ob* mice. **A** A Venn diagram shows the number of metabolites in response to *Mtb* infection in the blood of WT and *ob/ob* mice with *p* < 0.05. *WT* wild type. **B** A Venn diagram shows the number of metabolites in response to *Mtb* infection in the blood of wild type and *ob/ob* mice with *p* < 0.05 and FC > 1.5 or FC <  − 1.5. *FC* fold change. **C** Quantification of the one metabolite 3-aminoisobutyric acid in **A**. *p < 0.05. *ns* non-significant. **D** Quantification of the two common metabolites arginine and ornithine in **A**. *p < 0.05, **p < 0.01, ***p < 0.001

**Fig. 6:**
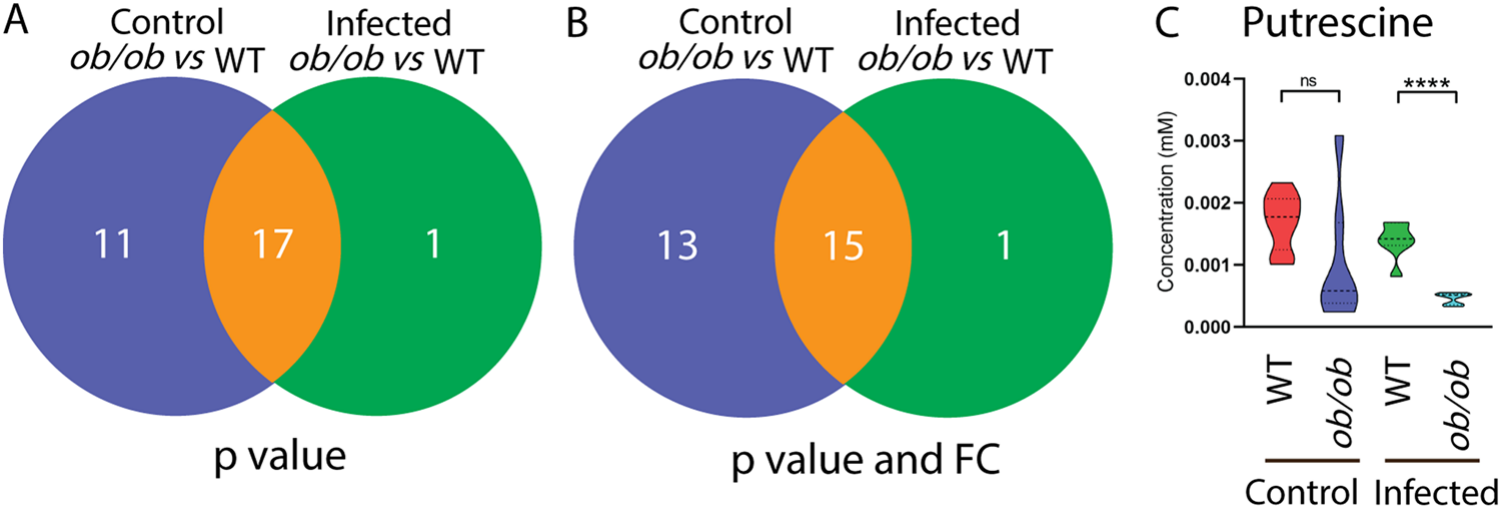
Venn diagrams show the number of metabolites measured by mass spectrometry between the blood of wild type and *ob/ob* mice in the uninfected control and infected conditions. **A** A Venn diagram shows the number of metabolites between the blood of WT and *ob/ob* mice in the uninfected control and infected conditions with *p* < 0.05. *WT* wild type. **B** A Venn diagram shows the number of metabolites between the blood of WT and *ob/ob* mice in the uninfected control and infected conditions with *p* < 0.05 and FC > 1.5 or FC <  − 1.5. *FC* fold change. **C** Quantification of the one metabolite putrescine in **A** and **B**. ****p < 0.0001. *ns* non-significant

Venn diagrams were generated to compare the metabolic effect of infection in the *ob/ob* mutant mice as compared to the WT control at a *p* value < 0.05 with and without applying a 1.5-FC filter. The Venn diagrams of Fig. 5A, B showed that the number of metabolites of which the levels were changed after infection in the *ob/ob* mutant mice was much lower than in the WT control. Only the level of one metabolite, 3-aminoisobutyric acid, was significantly changed in *ob/ob* mice but not in WT mice after infection (Fig. 5B). Although arginine levels were lower as a result of *Mtb* infection in the WT, they were higher in the *ob/ob* mice (Fig. 5C). After applying 1.5-FC filter, only one metabolite, namely ornithine, was commonly lower after infection in both WT and *ob/ob* mice (Fig. 5C). The Venn diagrams of Fig. 6A, B showed that the number of metabolites of which the levels were different between the *ob/ob* mutant and WT in the absence of infection was higher than in the presence of infection. This is because the concentrations of a majority of the metabolites were decreased already as a result of leptin mutation compared to WT mice. *Mtb* infection thus did not enhance the metabolic effects of leptin mutation. Only putrescine was significantly changed in *ob/ob* mice, but not in WT mice after infection (Fig. 6C). In conclusion, infection in the *ob/ob* mutant does not lead to lowering of the levels of the infection biomarker metabolites. Therefore, we conclude there is no clear synergy of the effects of leptin mutation and *Mtb* infection on metabolism. The leptin mutation, therefore, does not exacerbate the metabolic wasting syndrome caused by *Mtb* infection.

### The metabolic syndrome caused by leptin mutation and mycobacterial infection is similar in zebrafish and mice

We generated Venn diagrams to compare the metabolic effect of mycobacterial infection and leptin mutation in zebrafish measured by HR-MAS NMR spectroscopy and mice measured by MS (Supplementary Fig. 3). The result showed that leptin mutation leads to a similar metabolic syndrome as caused by mycobacterial infection in the two species, characterized by the decrease of 11 amine metabolites (Supplementary Fig. 3). The 11 common metabolites are alanine, citrulline, ethanolamine, glycine, histidine, isoleucine, leucine, methionine, phenylalanine, serine and threonine. Thus, the metabolic effects caused by leptin mutation and mycobacterial infection are highly conserved in zebrafish larvae and mice. Furthermore, we analyzed the changes of 22 common metabolites in zebrafish and mice in response to infection in the leptin mutant compared with the WT controls (Supplementary Table 3). By applying either a *p* value or a FC filter, nine metabolites were changed following the same pattern in zebrafish larvae and mice in response to infection in the leptin mutant and WT (Table 1). The nine metabolites include glycine, histidine, leucine, threonine, cysteine, methionine, asparagine, isoleucine, and tryptophan. There were four metabolites of which the level was no longer significantly changed in the leptin mutant after infection in both zebrafish larvae and mice (Table 1). The four metabolites were methionine, asparagine, isoleucine and tryptophan. We can conclude that in both species, the metabolic syndrome of the leptin mutant was not aggravated when the mutant was infected by mycobacteria.

**Table 1:**
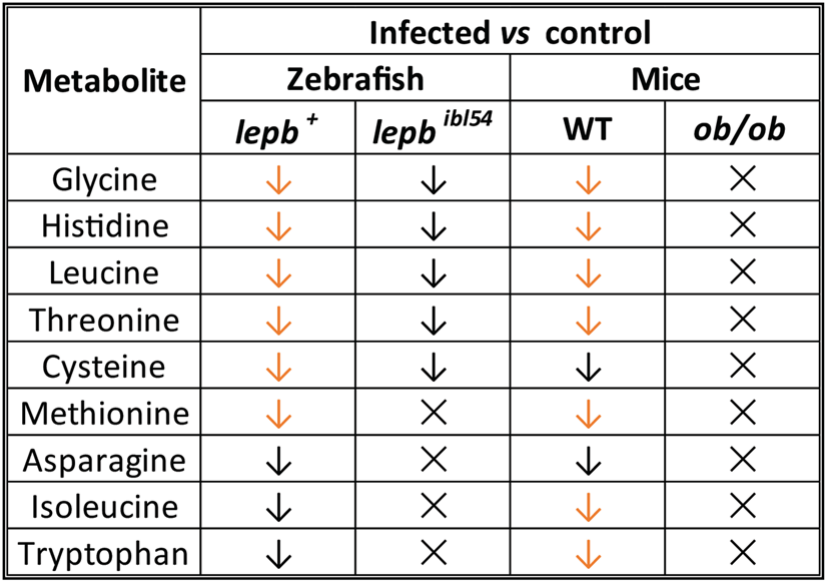
The changes of nine common metabolites in response to infection between the wild type and leptin mutant in zebrafish larvae and mice

↓ *p* < 0.05, downregulated, FC <  − 1.5; ↓ *p* < 0.05, downregulated, FC >  − 1.5; ✕ nonsignificant

### Deep sequencing of the transcriptomic response to infection of *lepb* mutant and wild type sibling zebrafish larvae

**Fig. 7:**
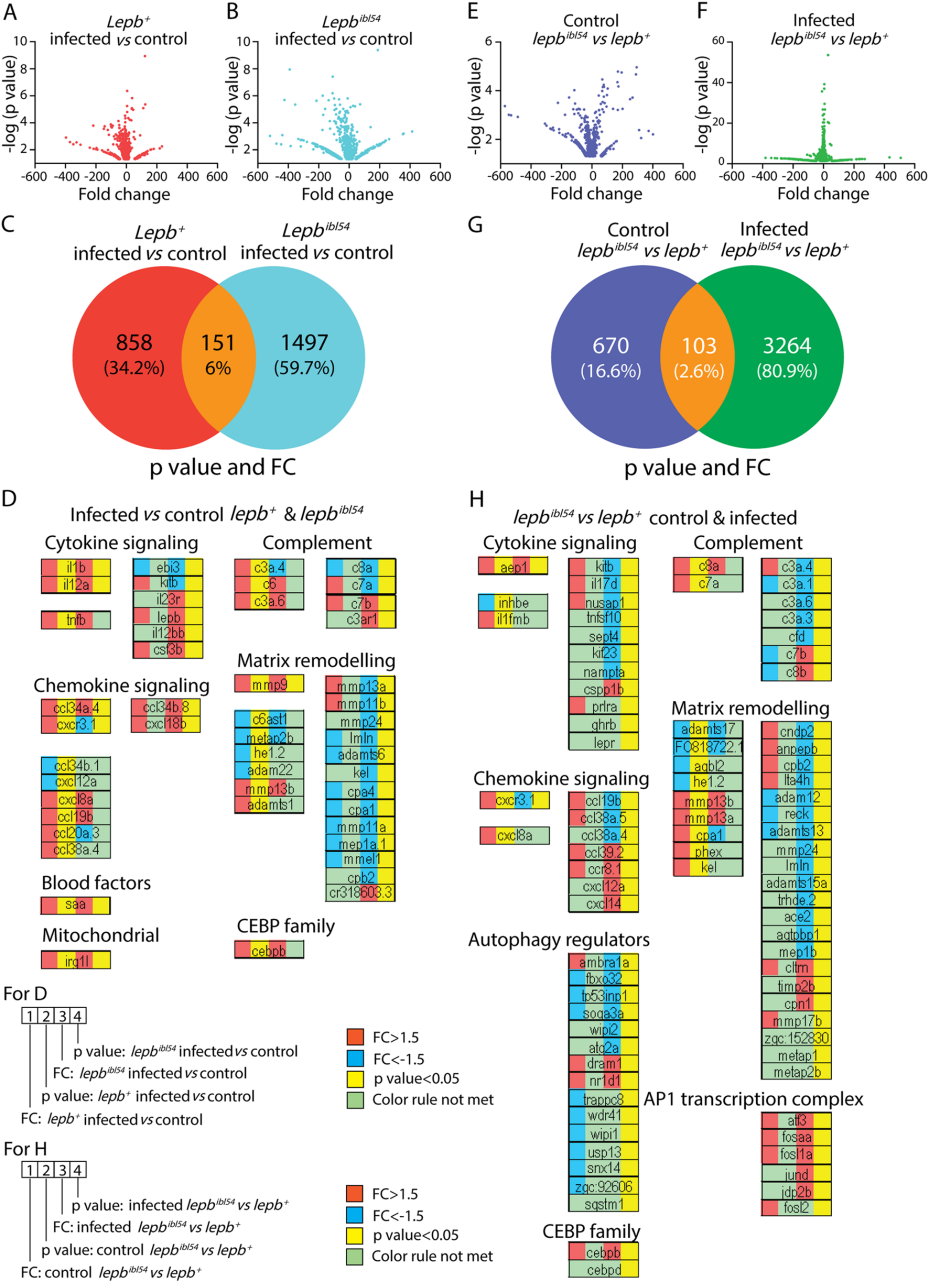
Transcriptome signature sets of *lepb*^*ibl54*^ and *lepb*^+^ zebrafish larvae at 5 dpi in the absence and presence of *M. marinum* infection. **A** A volcano plot of the signature set of *lepb*^+^ infected larvae compared to the *lepb*^+^ uninfected control. We used *p* < 0.05 and FC > 1.5 or FC <  − 1.5 as cutoff values for all the figures. **B** A volcano plot of the signature set of *lepb*^*ibl54*^ infected larvae compared to the *lepb*^*ibl54*^ uninfected control. **C** A Venn diagram shows the number of differentially expressed genes (DEGs) in response to infection in the *lepb*^+^ and *lepb*^*ibl54*^ larvae. **D** The FC and *p* value of the three groups of genes, shown in **C**, belonging to the two GO terms “response to bacterium” and “inflammatory responses”. **E** A volcano plot of the signature set of *lepb*^*ibl54*^ compared to *lepb*^+^ larvae in the uninfected control situation. **F** A volcano plot of the signature set of *lepb*^*ibl54*^ compared to *lepb*^+^ larvae in the infected situation. **G** A Venn diagram shows the number of DEGs between the *lepb*^*ibl54*^ and *lepb*^+^ in the uninfected control and infected conditions. **H** The FC and p value of the three groups of genes, shown in **G**, belonging to the two GO terms

We investigated the transcriptomic profiles of *lepb*^*ibl54*^ mutation and WT-siblings in the absence and presence of *M. marinum* infection in zebrafish larvae by RNAseq (Fig. 7A, B). Using significance cutoffs of *p* < 0.05 and 1.5-FC, the results showed that the mRNA levels of 1009 genes were significantly changed in *lepb*^+^ infected zebrafish larvae compared to the *lepb*^+^ uninfected control (Fig. 7C). Using the same *p* and FC cutoff values, the result showed that the mRNA levels of 1648 genes were significantly changed in *lepb*^*ibl54*^ infected zebrafish larvae compared to the *lepb*^*ibl54*^ uninfected control (Fig. 7C). The number of differentially expressed genes (DEGs) in the *lepb*^*ibl54*^ larvae in response to infection was therefore higher than in the *lepb*^+^ larvae (Fig. 7C). The Venn diagram of Fig. 7C showed 151 common genes in the two signature gene sets. GO enrichment analysis using database for annotation, visualization and integrated discovery (DAVID) resulted in significantly (*p* < 0.05) enriched GO terms for biological process of the three different groups in the Venn diagram of Fig. 7C (Supplementary Fig. 4A, B). The significantly enriched GO terms of the 151 common genes include “response to bacterium” and “inflammatory response” (Supplementary Fig. 4B). In Fig. 7D, we illustrated the FC and p value of the three groups of genes, shown in Fig. 3C, belonging to these two GO terms. A few genes, namely *il1b, il12a, cxl34a.4, saa, irg1l, mmp9* and *cebpb*, were significantly upregulated by infection in the common 151 signature set (Fig. 7D). More genes related to chemokine signaling were significantly changed in *lepb*^+^ compared to *lepb*^*ibl54*^ larvae after infection. However, the number of significantly regulated genes related to cytokine signaling, the complement cascade and matrix remodeling was higher in *lepb*^*ibl54*^ than in *lepb*^+^ larvae after infection (Fig. 7D).

**Fig. 8:**
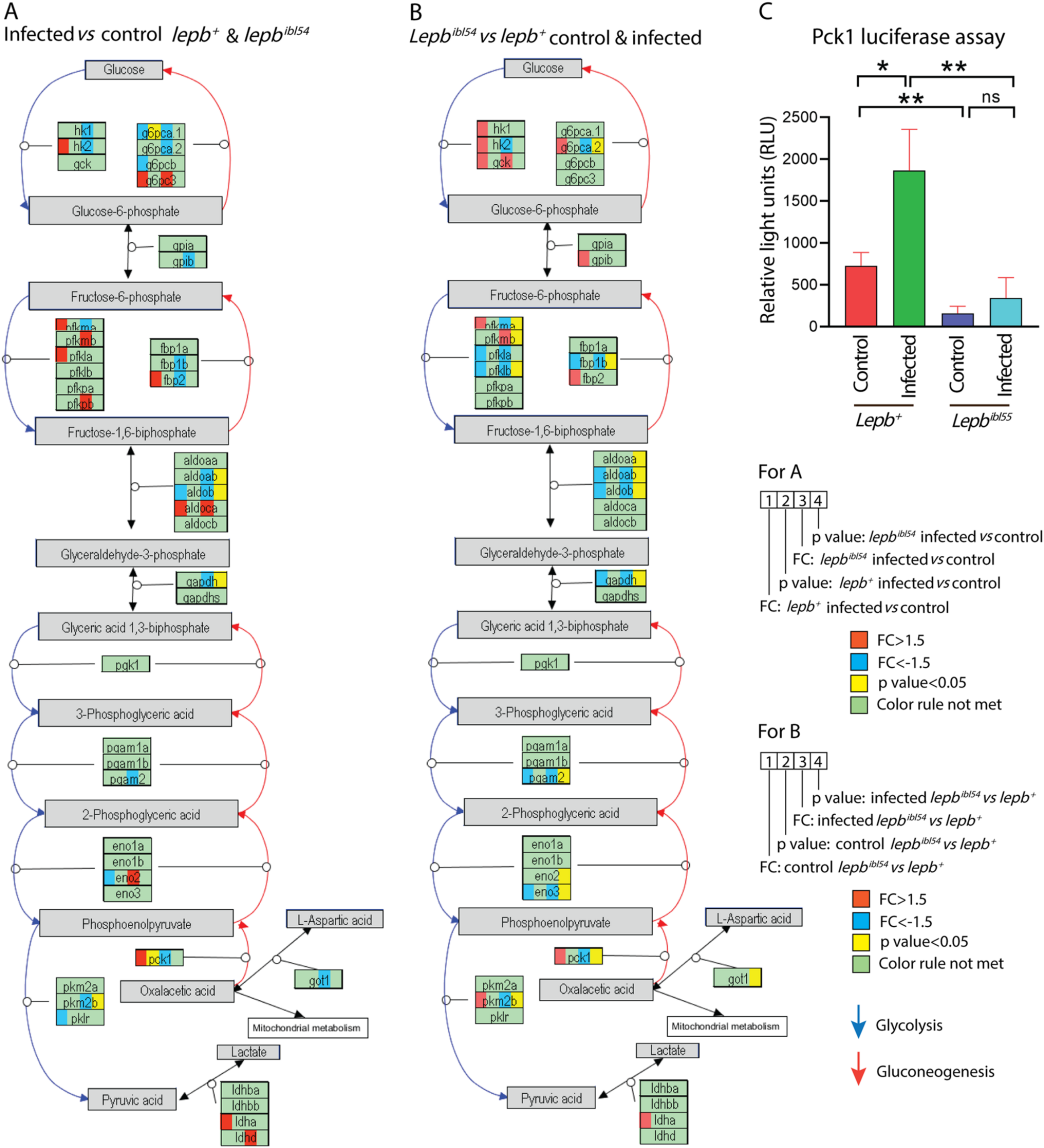
Genes regulated in glycolysis and gluconeogenesis pathway. **A** The DEGs in response to infection in the *lepb*^+^ and *lepb*^*ibl54*^ larvae. **B** The DEGs between the *lepb*^*ibl54*^ and *lepb*^+^ in the uninfected control and infected conditions. **C** The expression level of *pck1* in different groups detected by luciferase reporter assay (n = 24)

Next, we compared the number of genes that were differentially regulated between the mutant and the WT in the absence of infection (see volcano plot Fig. 7E) with the number of differentially regulated genes in the presence of infection (see volcano plot Fig. 7F). There were 773 genes differentially regulated in the absence of infection at *p* < 0.05 and 1.5-FC (Fig. 7G). However, there were 3367 genes differentially regulated at the same *p* value and FC cutoff in infected *lepb*^*ibl54*^ larvae compared to infected *lepb*^+^ siblings (Fig. 7G). The two signature sets encompassing 773 and 3367 genes showed an overlap of 103 genes (Fig. 7G). In Fig. 7H, we illustrated the FC and *p* value of the three groups of genes, shown in Fig. 7G, belonging to the two GO terms “response to bacterium” and “inflammatory response”. In the uninfected condition, there were 16 genes differentially regulated between the mutant and the WT with these GO terms, whereas in the infected condition there were 70 genes (Fig. 7H). In conclusion, the mutation of the *lepb*^*ibl54*^ gene and *M. marinum* infection cause synergistic effects in the transcription of inflammation related genes. We also performed the same comparisons with the genes in glycolysis and gluconeogenesis pathway because of the finding of the metabolomic analysis (Fig. 8). The result showed that only a few genes were differentially expressed after infection in both the mutant and the WT (Fig. 8A). *Pck1*, a key marker for gluconeogenesis, was significantly upregulated in *lepb*^+^ infected group, whereas it was not significantly changed in the *lepb*^*ibl54*^ infected group (Fig. 8A). In the uninfected condition, there were no genes differentially regulated between the mutant and the WT in this pathway, whereas in the infected condition there were 14 genes (Fig. 8B). In addition, *pck1* was significantly lower in the infected *lepb*^*ibl54*^ larvae compared to infected *lepb*^+^ larvae (Fig. 8B).

In order to measure *pck1* expression at high throughput level, we constructed transgenic derivatives of the *lepb*^*ibl55*^ mutant and WT control. Furthermore, we measured the expression level of the *pck1* with a luciferase reporter assay in the mutant and WT siblings in the absence and presence of infection (Fig. 8C). Consistent with the data from RNAseq, *pck1* expression was significantly increased upon infection in the WT, but not in the mutant larvae (Fig. 8C). In addition, the expression of *pck1* was also significantly lower in the infected *lepb*^*ibl55*^ in comparison with the *lepb*^+^ group (Fig. 8C). In conclusion, the results show that mycobacteria induce a very distinct transcriptome signature in the *lepb* mutant compared to the WT sibling control.

## Discussion

In this study, we studied the connections between the role of leptin in TB and T2DM by investigating the effects of mycobacterial infection in leptin deficient *lepb*^*ibl54*^ mutant zebrafish larvae and *ob/ob* mutant mice using metabolomics and transcriptomic techniques. We observed higher bacterial loads in the lungs of *ob/ob* mice infected with *Mtb* bacteria, compared to the WT infected controls (Fig. 4A). This observation is consistent with infection studies in *ob/ob* and leptin receptor (*db/db*) mutant mice. Wieland et al. (2005) observed a remarkably higher *Mtb* load in the lungs of *ob/ob* mice in comparison with the WT controls after 5 and 10 weeks of infection. Lemos et al. (2011) made the same observation in *db/db* mice infected with *Mtb*. Leptin signaling was also shown to play a key role in macrophage infection by other pathogens such as *Salmonella* Typhimurium (Fischer et al., 2019). However, in their study, leptin receptor (lepr) ablation reduced bacterial burden, suggesting that leptin signaling might play a different role in defense towards infections by different species of microbes. For our current study, we have used the zebrafish larval model infection system, which has the advantage of a non-feeding system in which the functional adaptive immune system is not yet present and therefore simplifies studies of the innate immune and metabolic responses to infection. Interestingly, we found that there are also significantly higher mycobacterial loads in the entire *lepb*^*ibl54*^ mutant zebrafish larvae compared to the WT siblings but not in the tail parts. This might be because leptin mutant zebrafish embryos have a different yolk composition that favors mycobacterial growth or an indirect metabolic systemic effect of this that could influence later stages of infection.

We found that leptin mutation and mycobacterial infection lead to a similar metabolic syndrome in zebrafish larvae as well as in mice (Table 1). This similarity could be explained by the occurrence of wasting syndrome in both the leptin mutants and during TB (Ding et al., 2020, 2021). In addition to wasting syndrome, *ob/ob* mice exhibit a phenotype of hyperglycemia, similar to human T2DM patients, indicating that leptin plays an important role in regulating glucose metabolism (Wang et al., 2014). Plasma leptin level is reduced in conditions of prolonged fasting (Sonnenberg et al., 2001) and leptin has been shown to be a key factor during starvation (Perry et al., 2018). In this study, we found that there are significantly higher glucose levels in the *lepb*^*ibl54*^ mutant zebrafish larvae as well as in the condition of *M. marinum* infection (Supplementary Table 1). *Pck1*, a marker of gluconeogenesis, was observed to be upregulated after infection in the WT but not in the *lepb*^*ibl54*^ mutant (Fig. 8A). Consistently, we found that *pck1* expression was lower in the infected *lepb*^*ibl54*^ mutant compared with the infected *lepb*^+^ siblings (Fig. 8B). These results were corroborated by using a *lepb*^*ibl55*^* Tg* (*pck1:luc1*) zebrafish line (Fig. 8C). The expression levels of several other genes in the infected group associated with the glycolysis pathway (Fig. 8B) were also found to be lower in the *lepb*^*ibl54*^ mutant than in the WT. Considering the lack of knowledge on the control of gluconeogenesis by infection and leptin signaling, it is hard to speculate on the relevance of these differences of the transcriptional responses of the *lepb*^*ibl54*^ mutant to infection. In any case, it is possible that the higher bacterial loads in the infected *lepb*^*ibl54*^ group lead to extreme limitations of carbon sources for gluconeogenesis and thereby might have triggered feed-back mechanisms. In support of this hypothesis, the decrease in levels of many glucogenic amino acids such as glycine, histidine, cysteine, methionine, asparagine, threonine, isoleucine and tryptophan in the *lepb*^*ibl54*^ mutant infected zebrafish larvae and *ob/ob* mutant infected mice indicates that the supply of the glucogenic amino acids in the mutants is limiting.

However, in both species, the decrease in the levels of amino acids is not aggravated in leptin mutants infected by mycobacteria. Interestingly, a metabolomic study using plasma from TB and TB–T2DM patients shows that the level of many metabolites such as citrulline, alanine, glutamine, ornithine, kynurenine and tryptophan is not aggravated in TB–T2DM patients compared with TB patients (Vrieling et al., 2019). We only see very few metabolites, namely trimethylamine N-oxide (Fig. 2E), mannose (Fig. 3C) in zebrafish larvae and 3-aminoisobutyric acid (Fig. 5C) and putrescine (Fig. 6C) in mice of which the levels are changed more severely in the leptin mutant in the presence of infection as compared to the WT. Therefore, we conclude that leptin and mycobacterial infection are non-synergistically controlling metabolism, but lead to a similar metabolic reprogramming. Nevertheless, it is possible that after infection of the leptin mutants as compared to the WTs, more energy is drained from long term storage supplies or depletion of muscle mass, as observed in severe wasting syndrome. This could explain the difference in responses of the *lepb*^*ibl54*^ mutant to infection found at the transcriptome level.

At the transcriptome level in zebrafish larvae, we observed that the number of genes of which the expression was significantly changed following *M. marinum* infection was higher in the *lepb*^*ibl54*^ mutant than in the sibling control (Fig. 7C). GO term enrichment analysis shows that in both the *lepb*^*ibl54*^ mutant and WT siblings, inflammatory responses to infection were highly enriched (Supplementary Fig. 4). However, there was a larger set of genes associated with inflammation responding to infection in the *lepb*^*ibl54*^ mutant than in the sibling control (Fig. 7D). This larger gene signature set includes genes of various cytokines, chemokines and genes involved in matrix remodeling and the complement cascade. When comparing the number of genes that are differentially expressed between the mutant and the WT in the absence of infection with the number of DEGs in the presence of infection, we also observe larger differences (Fig. 7E–G). In the absence of infection, the difference in transcriptional levels of inflammatory genes between the mutant and WT is very limited (Fig. 7G, H). In contrast, in the presence of infection many inflammatory genes have a much stronger response in the *lepb*^*ibl54*^ mutant than in the WT siblings. In addition to the cytokines, chemokines and genes involved in matrix remodeling and the complement cascade, we now also observe various genes of the AP1 transcription complex and genes involved in autophagy regulation to be stronger responding in the mutant (Fig. 7H). Different transcriptomic responses in leptin mutants and TB can lead to the similar metabolic end states. The distinction found in the transcriptomic profiles could either reflect a difference in the quality of the transcriptomic response between leptin mutant and WT larvae or reflect a difference in the kinetic of the response.

These data are in seeming contrast with the observations that leptin functions as a proinflammatory cytokine and plays a key role in immunity and inflammatory response in immune cells (Maurya et al., 2018a; Pérez-Pérez et al., 2020). Therefore, it has been used as an explanation why leptin deficiency leads to increased susceptibility of infection and it is a risk factor for many infectious diseases including TB (Maurya et al., 2018b). Our data shows that the function of leptin is very complex in that mutation of the *lepb* gene in an infection model leads to a very different signature set for inflammatory responses. Although there are common transcriptional responses that are still functional in the mutant, a particular set of response factors are selectively activated or inhibited. This is very different from what we found with the metabolic basic state and responses to infection in the mutant and WT.

In summary, it has been published that leptin deficiency increases susceptibility towards mycobacterial infection, impairs immune functions and dysregulates inflammatory responses (Iikuni et al., 2008; Lord et al., 1998; Maurya et al., 2018a). Many publications indicate that these effects of leptin deficiency could be due to a direct role in controlling cellular immunity. Our results confirm that there is a very different response in many inflammatory genes transcripts after infection in a zebrafish leptin mutant. However, the effect of the leptin mutation on the response to infection is very specific for a particular gene signature set and is not a general effect on all inflammatory genes. However, at the metabolism level, there is a general effect of the mutation on the levels of glucose and the glycolysis pathway and a pronounced function in metabolic reprogramming related to wasting syndrome. This effect of the mutation is highly similar to the effect of mycobacterial infection and is not synergistic. Therefore, we can conclude that the function of leptin in defense against mycobacteria is highly complex and is likely to be based on control of both inflammatory and system metabolism. Our metabolic and transcriptomic response signature sets of infection in the leptin mutant and WT controls can assist in the further study of the mechanisms underlying the role of leptin in glucose homeostasis, wasting syndrome and defense against infection. It thereby could provide further insights in the mechanisms of the connections between immunity and system metabolism that are still poorly understood.

## Conclusions

Leptin mutation leads to a similar metabolic syndrome as caused by mycobacterial infection in adult mice and larval zebrafish, characterized by the decrease of 11 amine metabolites. This observation is supported by different metabolomic technologies, namely MS and HR-MAS NMR spectrometer. In both species, this metabolic syndrome is not aggravated further when the leptin mutant are infected by mycobacteria. Therefore, we conclude that leptin and mycobacterial infection are both impacting metabolism non-synergistically. In addition, by studying the transcriptomes, it is shown that mycobacteria induced a very distinct transcriptome signature in the *lepb* mutant zebrafish compared to WT sibling control larvae. *Pck1* luciferase transgenic reporter lines are constructed and confirm this difference in transcriptional responses. Apparently, different transcriptomic responses in leptin mutants and TB can lead to similar metabolic end states.

## Supplementary Information

Below is the link to the electronic supplementary material.

Supplementary file1 (DOCX 1344 kb) **Supplementary Fig. 1** Representative ^1^H–^1^H COSY spectrum of 5 dpf embryo in the range of 0.5 to 5 ppm. The parameters used for COSY were 2048 data points collected in the t2 domain over the spectral width of 9k, 512 t1 increments were collected with 16 transients, relaxation delay 2 s, acquisition time 114 ms, and pre-saturated water resonance during relaxation delay. The resulting data were zero filled with 512 data points and were weighted with the squared sine bell window functions in both dimensions prior to Fourier Transformation. Application of gradient pulses along with tradition ^1^H–^1^H COSY sequence provides highly resolved spectrum. *Ala* alanine, *Arg* arginine, *Asp* aspartate, *Cho* choline, *Chol* cholesterol, *Cit* citrulline, *Cys* cysteine, *Eta* ethanolamine, *FA* fatty acid, *Glc* glucose, *Gln* glutamine, *Glu* glutamate, *GSH* glutathione, *His* histidine, *Ile* isoleucine, *Lac* lactate, *Leu* leucine, *Lys* lysine, *Met* methionine, *m-Ins* myo-inositol, *NAA N*-acetylaspartate, *Phe* phenylalanine, *PC* phosphocholine, *Pu* putrescine, *Ser* serine, *Tau* taurine, *Thr* threonine, *Trp* tryptophan, *Tyr* tyrosine. **Supplementary Fig. 2** Comparison of the number of biomarkers in intact and extracted zebrafish larvae due to *M. marinum* infection. A Venn diagram is shown of the overlap of the 20 metabolites of intact wild type zebrafish larvae after *M. marinum* infection measured by HR-MAS NMR in this study with the set of infection biomarkers in extracted zebrafish larvae measured by solution NMR published by Ding et al. (2020). **Supplementary Fig. 3** Common biomarkers for leptin mutation and mycobacteria infection in zebrafish larvae and mice. A Venn diagram shows that 11 common metabolites are significantly changed in both leptin mutation and mycobacteria infection in zebrafish larvae and mice. Common biomarkers of leptin mutation and infection in mice are from Ding et al. (2021). The 11 common metabolites are alanine, citrulline, ethanolamine, glycine, histidine, isoleucine, leucine, methionine, phenylalanine, serine and threonine. Three anti-correlated metabolites namely ATP, tyrosine and tryptophan are excluded. **Supplementary Fig. 4** A Venn diagram and GO terms. **A** A Venn diagram shows the number of differentially expressed genes (DEGs) in response to infection in the *lepb*^+^ and *lepb*^*ibl54*^ larvae with *p* < 0.05 and 1.5-FC. **B** Gene ontology (GO) enrichment analysis using DAVID resulted in significantly (*p* < 0.05) enriched GO terms for biological process of the three different groups in the Venn diagram of Supplementary Fig. S3A. **Supplementary Table 1** The fold change and *p* value of all the 35 quantified metabolites in the four different comparisons in zebrafish larvae. *ns* Non significant. *p < 0.05, **p < 0.01, ***p < 0.001, ****p < 0.0001. **Supplementary Table 2** The fold change and *p* value of all the 41 detectable metabolites in the four different comparisons in mice. *ns* non significant. *p < 0.05, **p < 0.01, ***p < 0.001, ****p < 0.0001. **Supplementary Table 3** The changes of 22 common metabolites in zebrafish and mice in response to infection in the wild types and leptin mutants. ↑p < 0.05, upregulated, FC < 1.5; ↓p < 0.05, downregulated, FC <  − 1.5; ↓p < 0.05, downregulated, FC > -1.5; ✕ non significant.

## Data Availability

All data generated or analyzed during this study are included in this published article and its Supplementary Information files.

## Abbreviations

DAVID: Database for annotation, visualization and integrated discovery
DEGs: Differentially expressed genes
dpf: Days post fertilization
dpi: Days post infection
FC: Fold change
GO: Gene ontology
HMDB: Human Metabolome Database
hpf: Hours post fertilization
HR-MAS NMR: High-resolution magic-angle-spinning NMR
Lepb: Leptin b
Lepr: Leptin receptor
*M. marinum*: *M* *ycobacterium marinum*
MS: Mass spectrometry
*Mtb*: *Mycobacterium tuberculosis*
NMR: Nuclear magnetic resonance
Pck1: Phosphoenolpyruvate carboxykinase 1
PLS-DA: Partial least squares discriminant analysis
RNAseq: RNA sequencing
T2DM: Type 2 diabetes mellitus
TB: Tuberculosis
TSP: Trimethyl-silylpropanoic acid
WT: Wild type
