# Supplementary material for: Leptin mutation and mycobacterial infection lead non-synergistically to a similar metabolic syndrome
Source: Metabolomics. 2022 Aug 7;18(8):67. doi: 10.1007/s11306-022-01921-8 (PMC9356939; doi:10.1007/s11306-022-01921-8)
Supplement: Supplementary file 1 — Supplementary file1 (DOCX 1344 kb) Supplementary Fig. 1 Representative 1H–1H COSY spectrum of 5 dpf embryo in the range of 0.5 to 5 ppm. The parameters used for COSY were 2048 data points collected in the t2 domain over the spectral width of 9k, 512 t1 increments were collected with 16 transients, relaxation delay 2 s, acquisition time 114 ms, and pre-saturated water resonance during relaxation delay. The resulting data were zero filled with 512 data points and were weighted with the squared sine bell window functions in both dimensions prior to Fourier Transformation. Application of gradient pulses along with tradition 1H–1H COSY sequence provides highly resolved spectrum. Ala alanine, Arg arginine, Asp aspartate, Cho choline, Chol cholesterol, Cit citrulline, Cys cysteine, Eta ethanolamine, FA fatty acid, Glc glucose, Gln glutamine, Glu glutamate, GSH glutathione, His histidine, Ile isoleucine, Lac lactate, Leu leucine, Lys lysine, Met methionine, m-Ins myo-inositol, NAA N-acetylaspartate, Phe phenylalanine, PC phosphocholine, Pu putrescine, Ser serine, Tau taurine, Thr threonine, Trp tryptophan, Tyr tyrosine. Supplementary Fig. 2 Comparison of the number of biomarkers in intact and extracted zebrafish larvae due to M. marinum infection. A Venn diagram is shown of the overlap of the 20 metabolites of intact wild type zebrafish larvae after M. marinum infection measured by HR-MAS NMR in this study with the set of infection biomarkers in extracted zebrafish larvae measured by solution NMR published by Ding et al. (2020). Supplementary Fig. 3 Common biomarkers for leptin mutation and mycobacteria infection in zebrafish larvae and mice. A Venn diagram shows that 11 common metabolites are significantly changed in both leptin mutation and mycobacteria infection in zebrafish larvae and mice. Common biomarkers of leptin mutation and infection in mice are from Ding et al. (2021). The 11 common metabolites are alanine, citrulline, ethanolamine, glycine, histidine, isoleucin [file 11306_2022_1921_MOESM1_ESM.docx]

**Supplementary Figure 1**

**
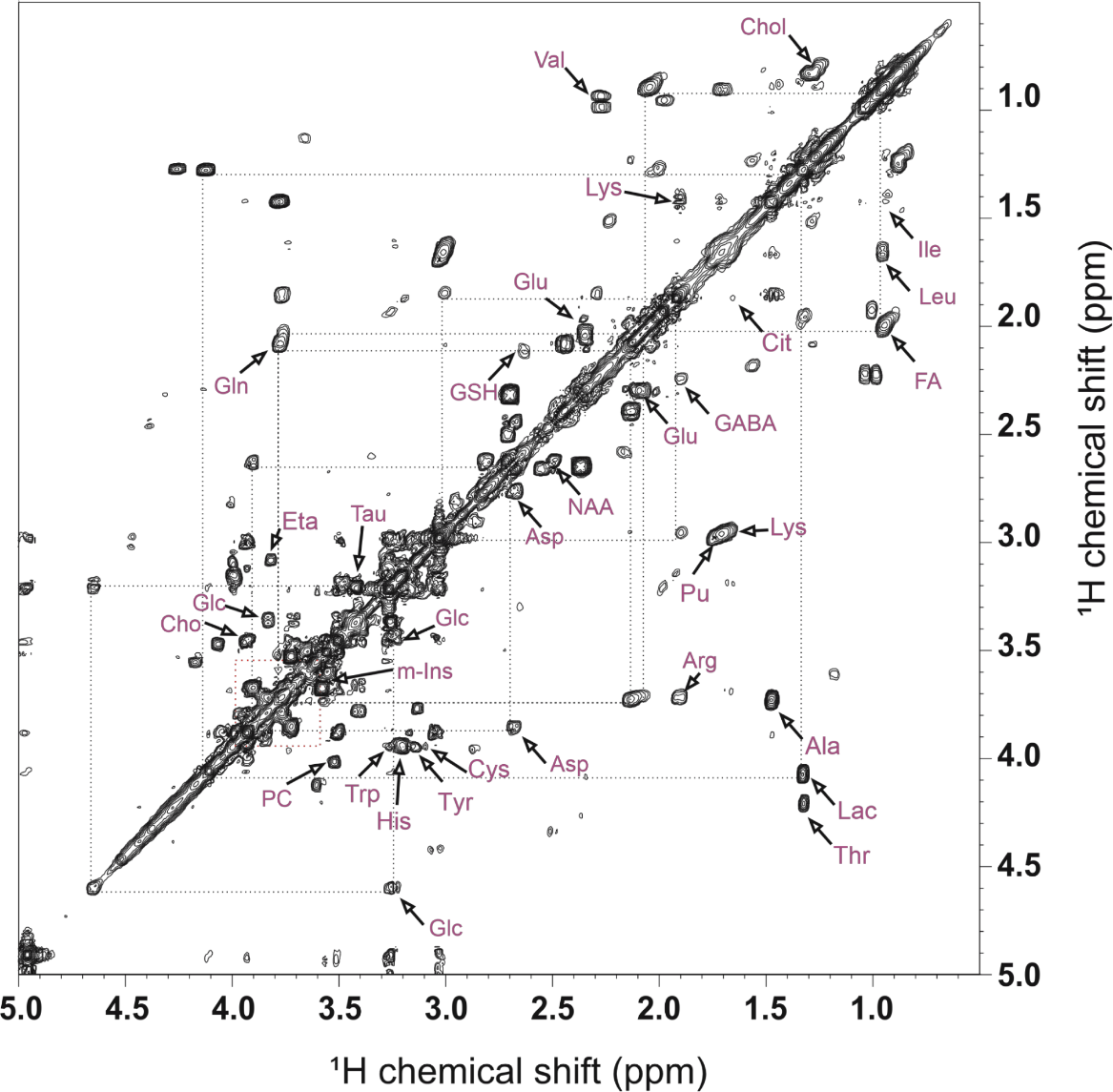
**

**Supplementary Figure 2**


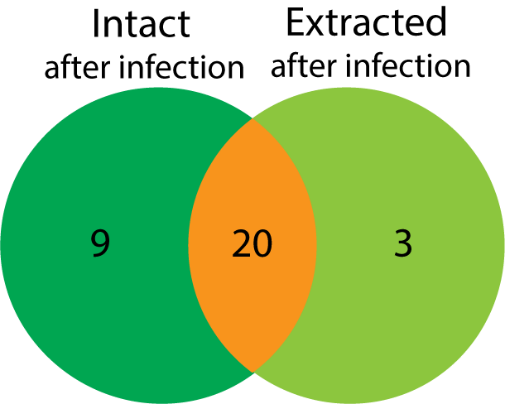


**Supplementary Figure 3**


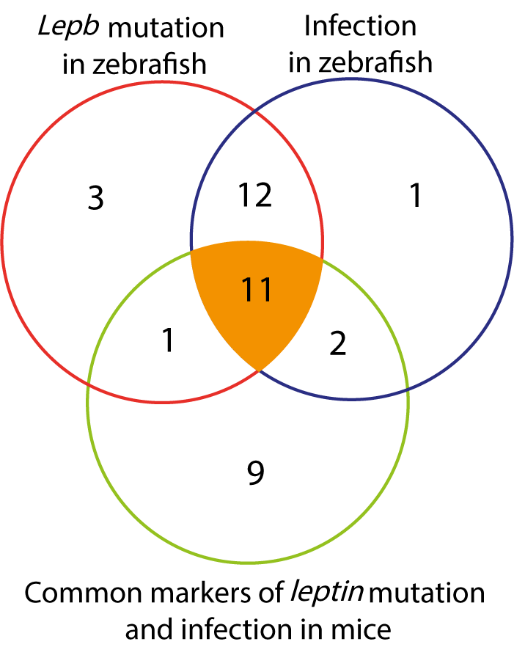


**Supplementary Figure 4**


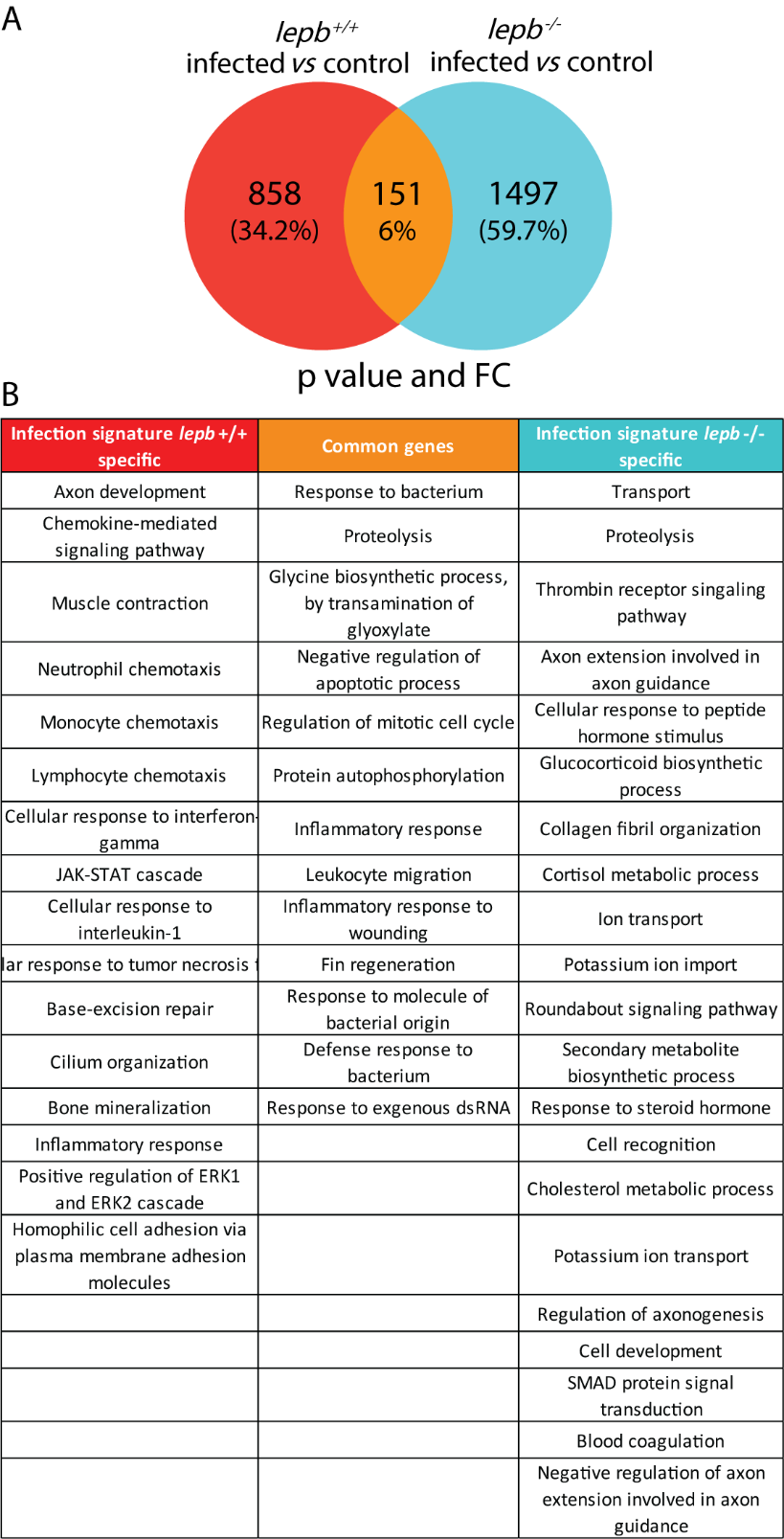


**Supplementary table S1**

**Supplementary table S2**

**Supplementary table S3**
